# Glabridin Mediate Caspases Activation and Induces Apoptosis through JNK1/2 and p38 MAPK Pathway in Human Promyelocytic Leukemia Cells

**DOI:** 10.1371/journal.pone.0098943

**Published:** 2014-06-05

**Authors:** Hsin-Lien Huang, Ming-Ju Hsieh, Ming-Hsien Chien, Hui-Yu Chen, Shun-Fa Yang, Pei-Ching Hsiao

**Affiliations:** 1 Institute of Medicine, Chung Shan Medical University, Taichung, Taiwan; 2 Cancer Research Center, Changhua Christian Hospital, Changhua, Taiwan; 3 School of Optometry, Chung Shan Medical University, Taichung, Taiwan; 4 Graduate Institute of Clinical Medicine, College of Medicine, Taipei Medical University, Taipei, Taiwan; 5 Wan Fang Hospital, Taipei Medical University, Taipei, Taiwan; 6 Department of Medical Research, Chung Shan Medical University Hospital, Taichung, Taiwan; 7 School of Medicine, Chung Shan Medical University, Taichung, Taiwan; 8 Department of Internal Medicine, Chung Shan Medical University Hospital, Taichung, Taiwan; Taipei Medical University, Taiwan

## Abstract

**Background:**

Glabridin, a prenylated isoflavonoid of *G. glabra L*. roots, has been associated with a wide range of biological properties such as regulation of energy metabolism, estrogenic, neuroprotective, anti-osteoporotic, and skin-whitening in previous studies. However, the effect of glabridin on tumor cells metastasis has not been clearly clarified. Here, the molecular mechanism by which glabridin anticancer effects in human promyelocytic leukemia cells was investigated.

**Methodology and Principal Findings:**

The results showed that glabridin significantly inhibited cell proliferation of four AML cell lines (HL-60, MV4-11, U937, and THP-1). Furthermore, glabridin induced apoptosis of HL-60 cells through caspases-3, -8, and -9 activations and PARP cleavage in dose- and time-dependent manner. Moreover, western blot analysis also showed that glabridin increase phosphorylation of ERK1/2, p38 MAPK and JNK1/2 in dose- and time-dependent manner. Inhibition of p38 MAPK and JNK1/2 by specific inhibitors significantly abolished the glabridin-induced activation of the caspase-3, -8 and -9.

**Conclusion:**

Taken together, our results suggest that glabridin induced HL-60 cell apoptosis through p38 MAPK and JNK1/2 pathways and could serve as a potential additional chemotherapeutic agent for treating AML.

## Introduction

Naturally occurring plant products have gained increasing attention for potential use in intervention against malignant invasive progression in late stage neoplastic diseases [1], [2]. There is increasing focus on providing a scientific basis for use of these agents as a preventive strategy for people with high risk of cancers. Glabridin as a key chemical and biological marker of G. glabra and have significant impact on food, dietary supplements (DSs) and cosmetic markets. Glabridin is an isoflavane, a type of isoflavonoid. This product is part of a larger family of plant-derived molecules, the natural phenols. The group’s previous study showed that glabridin can inhibit lung and breast cancer metastasis [3], [4]. Glabridin have shown positive effects in fields like LDL protection against oxidation and anti-obesity could potentially provide benefits to human health [5]–[7]. However, the effects of glabridin on human promyelocytic leukemia have yet to be evaluated.

Acute myeloid leukemia (AML), is a cancer of the myeloid line of blood cells, characterized by the rapid growth of abnormal white blood cells that accumulate in the bone marrow and interfere with the production of normal blood cells [8], [9]. Exposure to anticancer chemotherapy can increase the risk of subsequently developing AML. The risk is highest about three to five years after chemotherapy [10]. Conventional chemotherapy of AML combination treatment with cytarabine or daunorubicin induces complete remission in more than 50% of patients, only 20−30% of patients enjoy long-term disease-free survival and these chemo drugs can also affect normal cells causing unpleasant side effects such as anemia, bleeding, and infection. Thus, there is a need for new agents to treat AML. In recent years, some natural products have been used as alternative treatments for cancers including AML. Icariside II, a flavonoid compound derived from Epimedium koreanum, was suggested as an antileukemic agent for AML therapy [11]. Moreover, matrine, an alkaloid extracted from Sophora f lavescens Aif, targets mitochondrial apoptotic pathways in HL-60 [12]. Wogonin, an active compound in Scutellaria baicalensis, induces apoptosis by inhibiting telomerase activity in HL-60 [13]. In the present study, we investigated the cytotoxic effects of glabridin on HL-60 and its underlying mechanisms in vitro.

## Materials and Methods

### Chemicals

Glabridin, ≥98% (HPLC), powder was purchased from Sigma Chemical Co. (St. Louis, MO, USA). Stock solution of Glabridin was made at 10, 20 and 40 µM concentration in DMSO and stored at −20°C. The final concentration of DMSO for all treatments was consistently less than 0.1%. 3-(4,5-dimethylthiazol-2-y1)-2,5-diphenyltetrazolium bromide (MTT) was obtained from Sigma Chemical Co. (St. Louis, MO, USA). Specific inhibitors for ERK1/2 (U0126), JNK1/2 (SP600125) or p38 (SB202190) were purchased from Calbiochem (San Diego, CA). Antibodies, specifically of Bax, Bad, Bid, Bcl-2, cleaved caspase-3, caspase-8, caspase-9, poly (ADP-ribose) polymerase (PARP), p-extracellularly regulated kinase (ERK)1/2, p-p38, p-c-Jun N-terminal kinase (JNK), ERK1/2, p38, JNK1/2, α-tubulin and β-actin (for the Western blot analysis), were purchased from Santa Cruz Biotechnology.

### Cell Culture

Human AML cell lines of MV4-11 were kindly provided by Dr. L.-I. Lin (National Taiwan University, Taipei, Taiwan), while the HL-60, U937, and THP-1 cell lines were purchased from the American Type Culture Collection (ATCC) (Manassas, VA). Human normal cells of WI-38 (lung fibroblast) and HOK (human oral keratinocyte cell) were purchased from ATCC. All cells were cultured in the recommended conditions, supplemented with 10% fetal bovine serum (FBS), 0.1 mM nonessential amino acids, 1 mM glutamine, 1% penicillin/streptomycin, 1.5 g/L sodium bicarbonate, and 1 mM sodium pyruvate (Sigma, St. Louis, Mo, USA) and maintained at 37°C in a humidified atmosphere of 5% CO2.

### In vitro Cytotoxicity Assay

The effect of glabridin on cell growth was assayed by the MTT (3- (4,5-cimethylthiazol-2-yl)-2,5-diphenyl tetrazolium bromide) method, as previously described [14]. Briefly, cells were cultured in 24-well plates (5×10^4^/well) and stimulated with different concentrations of glabridin (0, 10, 20, 40, 80 µM) in culture media. After 24 hours of glabridin stimulation, MTT was added to each well (0.5 mg/ml final concentration) with a further incubation for 4 h. The viable cell number was directly proportional to the production of frmazan following the solubilization with isopropanol. The color intensity was measured at 570 nm [15]. Each condition was performed in triplicate and data were obtained from at least 3 separate experiments.

### Cell Cycle Analysis

To determine the effect of glabridin on cell cycle, cells (1×10^6^/ml) were first cultured in serum-free medium for starvation at 18 hours and then exposed to glabridin for 24 hours. After cells were washed, fixed with 70% ethanol, and incubated for 30 minutes in the dark at room temperature with propidium iodide (PI) buffer (4 µg/ml PI, 1% Triton X-100, 0.5 mg/ml RNase A in PBS) and then filtered through a 40-µm nylon filter (Falcon, USA) [16]. The cell cycle distribution was analyzed for 5,000 collected cells by a FACS Vantage flow cytometer that uses the CellQuest acquisition and analysis program (Becton Dickinson FACSCalibur).

### Annexin V/PI Double Staining

To detect apoptosis in HL-60 cell line after exposure to glabridin, an FITC Annexin V Apoptosis Detection Kit I (BD Biosciences, USA) was used to quantify cell number in different stages of cell death [17]. Briefly, 1×10^5^ cells were resuspended in 100 µl 1× binding buffer (0.01 M Hepes/NaOH (pH 7.4), 0.14 M NaCl, 2.5 mM CaCl2). After the addition of FITC Annexin V and PI (5 µl each), the cell suspension was gently vortexed and incubated for 15 minutes at room temperature in the dark. Add 400 µl of 1× binding buffer to each tube and analyze by flow cytometry within 1 h [18].

### Western Blot Analysis

Cell lysates were separated in a 10% or 15% polyacrylamide gel and transferred onto a PVDF membrane (Millipore Corporation, Milford, MA, USA) [19]. The blot was subsequently incubated with 5% non-fat milk in PBS for 1 h to block non-specific binding, and probed with a corresponding antibody against a specific protein for 37°C at 2 h or overnight at 4°C, and then with an appropriate peroxidase conjugated secondary antibody for 1 h. After the final washing, signal was developed by ECL detection system and relative photographic density was quantitated by a gel documentation and analysis (AlphaImager 2000, Alpha Innotech Corporation, San Lean 189 dro, CA, USA) [20].

### Statistical Analysis

Values represent the means ± standard deviation and the experiments were repeated three times (n = 3). Statistical analyses were performed using the one-way analysis of variance (ANOVA) followed by Tukey’s posthoc test was used when more than three groups were analyzed. Data comparisons were performed with Student’s t test (Sigma-Stat 2.0, Jandel Scientific, San Rafael, CA, USA) when two groups were compared. A p value <0.05 was considered to be statistically significant.

## Results

### Cytotoxic Effects of Glabridin-treated AML Cell Lines

**Figure 1:**
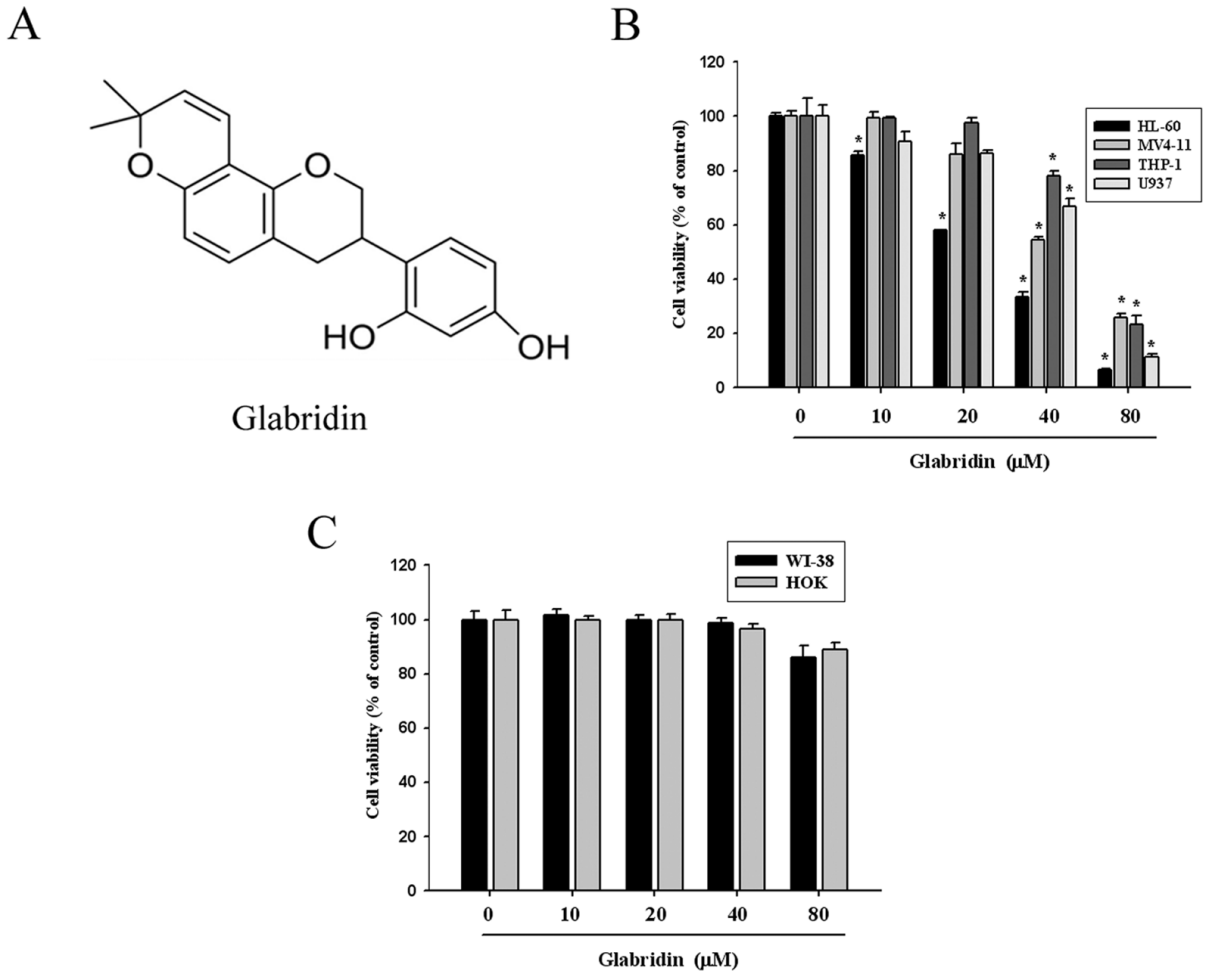
Cytotoxic effect of glabridin in four AML cell lines (HL-60, MV4-11, U937, and THP-1) and two human normal cells (WI-38 and HOK). (A) Structure of glabridin. (B) Cell viability analysis of four AML cell lines (HL-60, MV4-11, U937, and THP-1) cultured in presence of glabridin for 24 h by MTT assay. (C) Cell viability analysis of two normal cells (WI-38 and HOK) cultured in presence of glabridin for 24 h by MTT assay. Data represent mean of 3 determinations per condition repeated 3 times. Results are shown as mean ± SE.

The chemical structure of glabridin was shown in Figure 1A. To determine the cytotoxicity and the effect on cell proliferation of glabridin on four AML cell lines (HL-60, MV4-11, U937, and THP-1), cells were treated with different concentrations of glabridin (0–80 µM) for 24 h. As shown in Figure 1B, after treatment for 24 h, glabridin significantly reduced the cell viability in a concentration-dependent manner for the four AML cell lines. Moreover, glabridin did not alter the cell viability of normal lung fibroblast cells (WI-38) and human oral keratinocyte cell (HOK) (Figure 1C).

### Glabridin-induced Cell Apoptosis in HL-60 and MV4-11 Cells

**Figure 2:**
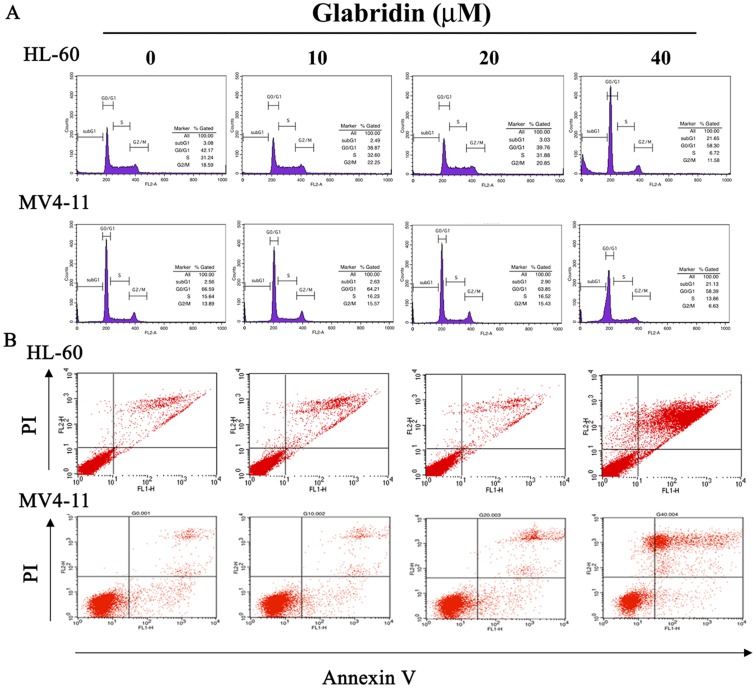
Glabridin induced cell apoptosis in HL-60 and MV4-11 cells. (A) HL-60 and MV4-11 cells were incubated for 18 h in the absence of serum and then treated with indicated concentrations of glabridin for 24 h, after which the cells were stained with PI, and analyzed for DNA content by flow cytometry. Furthermore, after being treated with different concentration of glabridin for 24 h, cells were harvested and then subjected to quantitative analysis of cell apoptosis by Annexin-V and PI double-stained flow cytometry (B).

To determine whether the inhibitory effect of cell viability of glabridin is associated with induction of cell apoptosis, HL-60 and MV4-11 cells were treated with different concentrations (0–40 µM) of glabridin for 24 h. Cell cycle analysis by flow cytometry was showed a dose-dependent increased accumulation of cell population in sub-G1 phase after a 24-hour treatment with glabridin (Figure 2A). Meanwhile, Annexin-V and PI double-staining displayed an increased percentage of apoptotic cells after a 24 h treatment of glabridin (Figure 2B), respectively.

### Glabridin Induced Activation of Caspase-3, -8 and -9 in HL-60 Cells

**Figure 3:**
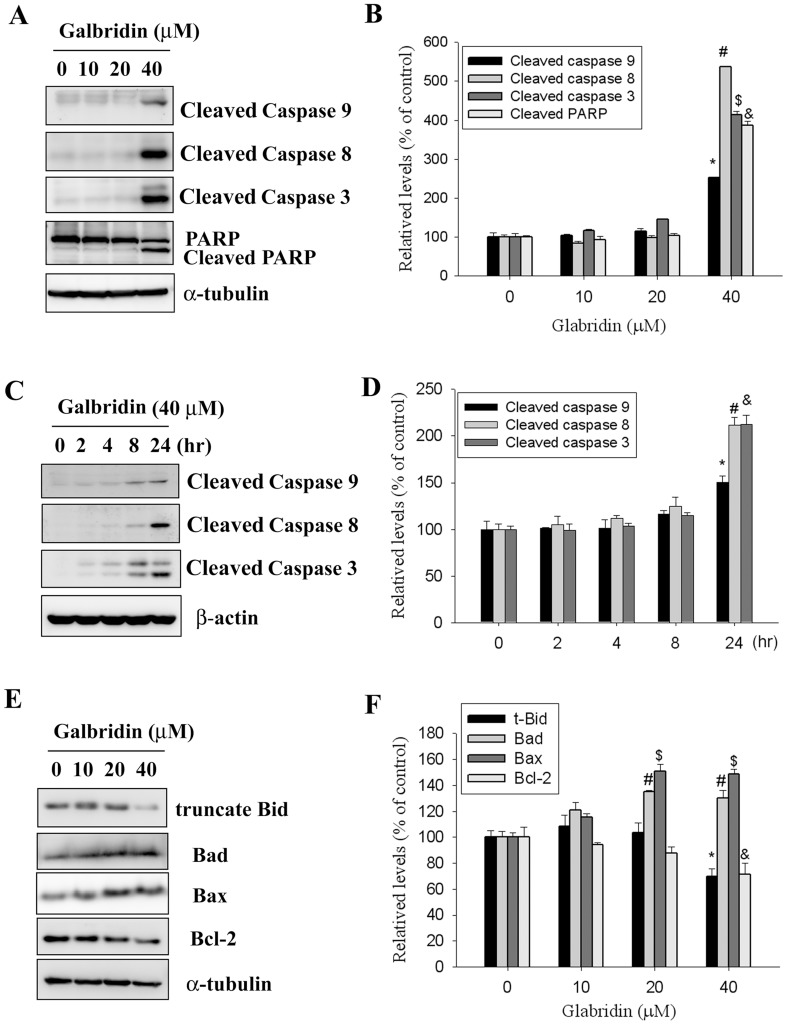
Activation of caspase 3, -8, -9 and PARP were increased in Glabridin-treated HL-60 cells. (A) HL-60 cells were treated with 10, 20 and 40 µM glabridin for 24 h and subjected to western blotting with an antibody against PARP or caspase-3, -8 and -9 antibody. (B) The values under each lane indicate relative density of the band normalized to α-tubulin using a densitometer. Values represent the mean ± SE of three independent experiments. (*, #, &, $) p<0.05 compared to the vehicle control groups. (C) HL-60 cells were treated with 40 µM glabridin for 2, 4, 8 and 24 h, subjected to western blotting with an antibody against caspase-3, -8 and -9 antibody. (D) The values under each lane indicate relative density of the band normalized to β-actin using a densitometer. Values represent the mean ± SE of three independent experiments. (*, #, &) p<0.05 compared to the vehicle control groups. (E) HL-60 cells were treated with 10, 20 and 40 µM glabridin for 24 h and subjected to western blotting with an antibody against Bid, Bax, Bad and Bcl-2 antibody. (F) The values under each lane indicate relative density of the band normalized to α-tubulin using a densitometer. Values represent the mean ± SE of three independent experiments. (*, #, &, $) p<0.05 compared to the vehicle control groups.

To further confirm the involvement of caspase activation in glabridin-induced apoptosis, activation of caspases-3, -8, and -9 and cleavage of PARP were detected. Figure 3A shows that exposure of HL-60 cells to glabridin (0–40 µM) for 24 h, caused concentration-dependent increased of the cleaved fragments of caspases-9, -8, and -3. Furthermore, cleaved PARP was also significantly increased in glabridin treated HL-60 cells. Glabridin treatment at 40 µM for 24 h significantly increased expression levels of cleaved caspases-3, -8, -9 and PARP by 4.1-, 2.37-, 5.37 and 3.87-fold, respectively, compared to the control (Figure 3B). Moreover, treatment of HL-60 cells with glabridin (40 µM) also resulted in a time-dependent increase in activated caspases-3, -8, and -9 (Figure 3C). Glabridin treatment at 40 µM for 24 h significantly increased expression levels of cleaved caspases-3, -8 and -9 by 2.12-, 2.11- and 1.5-fold, respectively, compared to the control (Figure 3D). Glabridin caused concentration-dependent decreases in the truncate Bid and Bcl-2, in addition, the expression levels of Bax and Bad were increases in glabridin-treated HL-60 cells (Figure 3E). Glabridin treatment at 40 µM for 24 h significantly increased expression levels of Bad and Bax by 1.3 and 1.48-fold, it also decreased expression levels of truncate Bid and Bcl-2 by 29% and 31%, respectively, compared to the control (Figure 3F).

### The Apoptosis Induction by Glabridin is Dependent on the Regulation of JNK and P38 MAPK Signaling Pathways in HL-60 Cells

**Figure 4:**
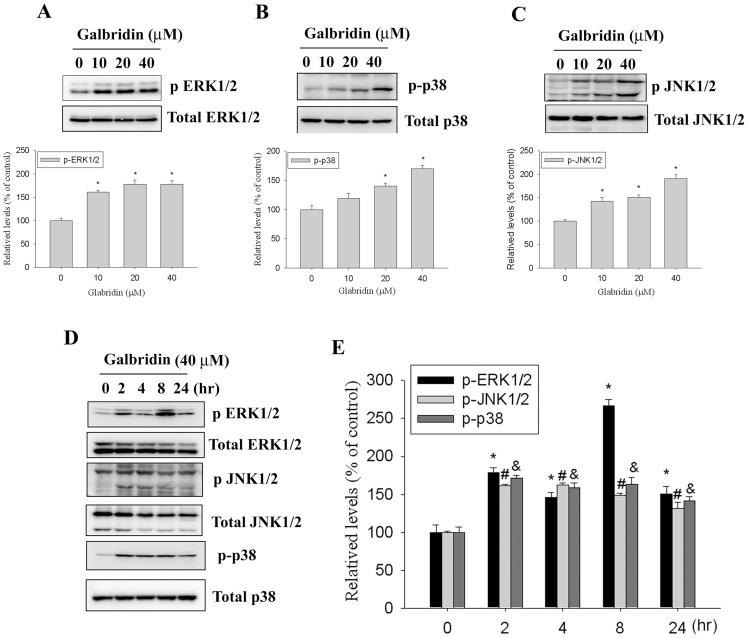
Glabridin activates the phosphorylation of ERK1/2, p38 MAPK and JNK1/2 in HL-60 cells. (A–C, upper panel)) Cells were treated with different concentrations of glabridin (0–40 µM) for 24 h and then subjected to western blotting with an antibody against ERK1/2, JNK1/2, and p38 MAPK. (A–C, lower panel) Quantitative results of p-ERK1/2, p-p38, and p-JNK1/2 protein levels, which were adjusted with the total ERK1/2, p38, and JNK1/2 protein levels and expressed as multiples of induction beyond each respective control. Values represent the mean ± SE of three independent experiments. (*) p<0.05 compared to the vehicle control group. (D) HL-60 cells were treated with 40 µM glabridin for 2, 4, 8 and 24 h, subjected to western blotting with an antibody against ERK1/2, JNK1/2, and p38 MAPK antibody. (E) The values under each lane indicate relative density of the band normalized to β-actin using a densitometer. Values represent the mean ± SE of three independent experiments. (*, #, &, $) p<0.05 compared to the vehicle control groups.

**Figure 5:**
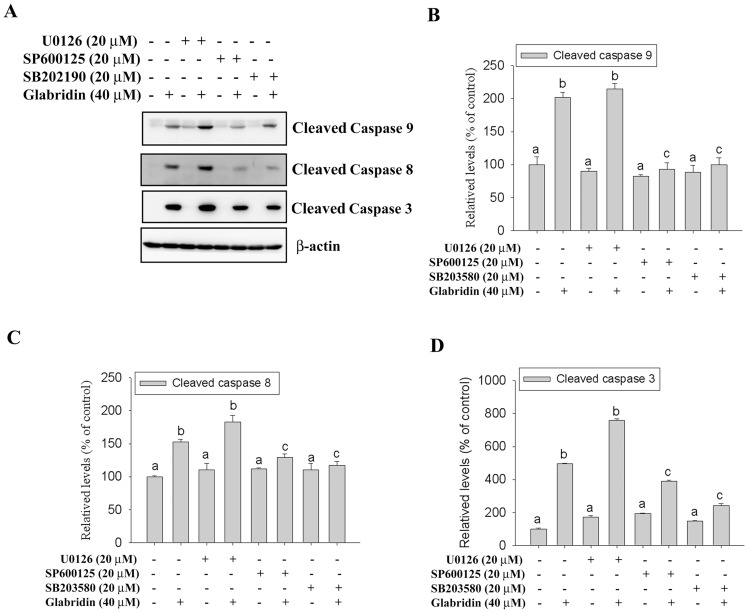
JNK1/2 and p38 MAPK are essential for caspase activation induced by glabridin. (A) HL-60 cells were treated for 24 hours with 40 µM glabridin with or without a 1-hour pretreatment of 20 µM U0126, SP600125, or SB202190. The expression of cleaved caspase-9, -8 and -3 were detected by western blotting. (B–D) Quantitative results of cleaved caspase-9, -8, and -3 protein levels, which were adjusted to the β-actin protein level and expressed as multiples of induction beyond each respective control. Values represent the mean ± SE of three independen experiments. Data were analyzed using a one-way ANOVA with Tukey’s posthoc tests at 95% confidence intervals; different letters represent different levels of significance.

Several signaling pathways have been implicated MAPK signaling pathway plays an important role in the action of chemotherapeutic drugs in the regulation of apoptosis [21]. In a further investigation for the underlying molecular mechanisms, we determined whether MAPKs were activated in glabridin-treated HL-60 cells by a Western blot analysis. Results showed that the phosphorylation of ERK1/2, JNK1/2 and p38 MAPK were increased in cells treated with glabridin in a dose-dependent manner (Figure 4A, 4B and 4C). Moreover, treatment of HL-60 cells with glabridin (40 µM) also resulted in a time-dependent increase in phosphorylation of ERK1/2, JNK1/2 and p38 MAPK (Figure 4D and 4E). Next, we further investigated relationships among glabridin induced activation of caspases-9, -8, and -3 and MAPKs. HL-60 cells were pretreated with 20 µM U0126 (an ERK inhibitor), SP600125 (a JNK inhibitor), or SB202190 (a p38 inhibitor) for 1 h, treated with 40 µM glabridin for another 24 h, and then analyzed by Western blotting. As shown in Figure 5, treatment SB202190 and SP600125 significantly attenuated glabridin-induced caspase-9, -8, and -3 activation. These findings suggest that activation of JNK1/2 and p38 MAPK might play a critical upstream role in glabridin-mediated caspase activation in HL-60 cells.

## Discussion

Acute myeloid leukemia (AML) is a fatal hematological malignancy which is resistant to a variety of chemotherapy drugs. Natural herbal products have a promising and potential role in developing novel chemotherapeutics for various cancers [22]–[24]. Various ethnic societies worldwide have traditionally used herbal products in the prevention and/or treatment of several chronic diseases [25]–[27]. Glabridin is a polyphenolic flavonoid, a main constituent in the roots of *Glycyrrhiza glabra* possesses various biological activities, has reported protection against oxidation, anti-obesity and inhibit lung and breast cancer metastasis [4], [28], [29]. Glabridin being a natural product and with known safety in human is a suitable candidate for drug development [30]. The present study demonstrates, for the first time, that glabridin induced apoptosis of HL-60 cells.

Apoptosis, is a form of programmed cell death that occurs naturally in cells and can be beneficial to cancer therapy as previously reported [31], [32]. Natural herbal products have been extensively studied for its antitumor effect including antiproliferative activities, cell cycle arrest and apoptosis induction via the mitochondrial and some other pathways [33], [34]. Recently, targeted elimination of AML cells by inducing apoptosis has emerged as a valuable strategy for combating AML [12], [35]. Moreover, several naturally occurring drugs such as homoharringtonine and etoposide are used in the clinic for treating AML [36]. In this study, several hallmarks of apoptosis such as significant increases in sub-G1 content, and Annexin V-positive cells were observed in HL-60 cells after glabridin treatment for 24 h. Glabridin (40 µM) can induce increases of sub-G1 content and Annexin V-positive cells by 6.7- and 8.2-fold, respectively.

Apoptosis is mediated by proteolytic enzymes called caspases, which trigger cell death by cleaving specific proteins in the cytoplasm and nucleus. The activation process is initiated by either extracellular or intracellular death signals, which cause intracellular adaptor molecules to aggregate and activate procaspases [37]. The present results suggest that glabridin may partially act through the initiator caspase-8 and then the executioner caspase-3 to increase the cleavage form of PARP to induce AML cell apoptosis. Moreover, many papers pointed out that the ability of anticancer agents to induce apoptosis of tumor cells was correlated with the ability to decrease expression of Bcl-2 [38]. Meanwhile, we found that expression of the antiapoptotic proteins Bcl-2 also was decreased in HL-60 cells after glabridin treatment for 24 h. Other members of the Bcl-2 family are not death inhibitors, but instead promote procaspase activation and cell death. Some of these apoptosis promoters, such as Bad, function by binding to and inactivating the death-inhibiting members of the family, whereas others, like Bax and Bak, stimulate the release of cytochrome *c* from mitochondria. Bax and Bak are themselves activated by other apoptosis-promoting members of the Bcl-2 family such as Bid [39]. In the present study, an increase in the Bad and Bax protein expression levels, a decrease in the expression of Bid, and activation of caspases-3/−9 occurred after treatment with glabridin, suggesting that glabridin inducing apoptosis in HL-60 cells might partly occur through a mitochondrion-mediated pathway.

Previous studies have suggested that MAPKs can be induced by various compounds and involved in cell death or cytoprotection in AML cells [40]–[42]. On the basis of previous reports, we further investigated activation of MAPK family proteins in glabridin treated HL-60 cells. The results showed that the phosphorylation of ERK1/2, JNK1/2, and p38 MAPK were increased in glabridin-treatment HL-60 cells and glabridin (40 µM) induced activation of ERK1/2, JNK1/2, and p38 MAPK in a time-dependent manner. However, treatment with JNK specific inhibitor (SP600125) or p38 MAPK specific inhibitor (SB202190) effectively inhibit activation of caspases-3, -8, and -9 induced by glabridin, whereas U0126 (an ERK1/2 inhibitor) had no effect on glabridin-induced caspase activation. Taken together, these results suggest that activation of JNK1/2 and p38 MAPK plays an important role in glabridin-induced apoptosis.

In conclusion, we first demonstrated that glabridin could induce the phosphorylation of JNK1/2, and p38 MAPK, inhibit the expressions of Bcl-2 and Bid, subsequent stimulate the activation of caspase-3, -8, and -9, which eventually result in the cleaved of PARP and inhibition of proliferation and apoptosis induction of HL-60 cells. Our findings revealed that glabridin may be a useful candidate as a chemotherapeutic agent for AML therapy.
